# Ontology-guided data preparation for discovering genotype-phenotype relationships

**DOI:** 10.1186/1471-2105-9-S4-S3

**Published:** 2008-04-25

**Authors:** Adrien Coulet, Malika Smaïl-Tabbone, Pascale Benlian, Amedeo Napoli, Marie-Dominique Devignes

**Affiliations:** 1 KIKA Medical, Paris, F-75012, France; 2 LORIA (UMR 7503 CNRS-INPL-INRIA-Nancy2-UHP), Vandoeuvre-lès-Nancy, F- 54506, France; 3 Université Pierre et Marie Curie - Paris6, INSERM UMRS 538 Biochimie-Biologie Moléculaire, Paris, F-75571, France

## Abstract

**Background:**

Complexity and amount of post-genomic data constitute two major factors limiting the application of Knowledge Discovery in Databases (KDD) methods in life sciences. Bio-ontologies may nowadays play key roles in knowledge discovery in life science providing semantics to data and to extracted units, by taking advantage of the progress of Semantic Web technologies concerning the understanding and availability of tools for knowledge representation, extraction, and reasoning.

**Results:**

This paper presents a method that exploits bio-ontologies for guiding data selection within the preparation step of the KDD process. We propose three scenarios in which domain knowledge and ontology elements such as subsumption, properties, class descriptions, are taken into account for data selection, before the data mining step. Each of these scenarios is illustrated within a case-study relative to the search of genotype-phenotype relationships in a familial hypercholesterolemia dataset. The guiding of data selection based on domain knowledge is analysed and shows a direct influence on the volume and significance of the data mining results.

**Conclusions:**

The method proposed in this paper is an efficient alternative to numerical methods for data selection based on domain knowledge. In turn, the results of this study may be reused in ontology modelling and data integration.

## Background

The Knowledge Discovery in Databases (KDD) process is based on three main operations: data preparation, data mining, and interpretation of the extracted units. This process is guided and controlled by an expert of the concerned domain. The KDD process has been successfully applied in various domains such as marketing, finance, and biomedicine [1].

However applications of KDD are limited by the fact that strong interactions between the system and domain experts are necessary. Data manipulated in life sciences are complex and data mining algorithms generate large volume of rough results. As a consequence, the interpretation step of KDD in biology, aimed at extracting new and relevant knowledge units, is a hard task, i.e. time-consuming and tedious for the domain expert.

In computer science, ontologies provide a shared understanding of knowledge about a particular domain [2]. Bio-ontologies are becoming more and more available and contribute to the understanding of the large amounts of data existing in life sciences [3]. The National Center for Biomedical Ontology (NCBO) has recently developed Bioportal that offers a unified panorama on available bio-ontologies [4,5].

One of the promising interests of bio-ontologies is their use for guiding the process of KDD as suggested by Anand [6], Cespivova [7], Gottgtroy [8], and Napoli [9]. This idea seems to be much more realistic now that Semantic Web advances have given rise to common standards and technologies for expressing and sharing ontologies [10].

In this way, the three main operations of KDD can take advantage of domain knowledge embedded in bio-ontologies.

(1) During the data preparation step, bio-ontologies can facilitate the integration of heterogeneous data and guide the selection of relevant data to be mined.

(2) During the mining step, domain knowledge allows the specification of constraints for guiding data mining algorithms by, e.g. narrowing the search space.

(3) During the interpretation step, domain knowledge helps experts to visualize and validate extracted units.

There exists a number of studies on the use of ontologies within the data mining step, e.g. [11,12], and the interpretation step e.g. [13-15]. Only a few studies (detailed hereafter) has focused on the first step, namely data preparation. This is the purpose of the present paper.

Data preparation –or preprocessing– is aimed at improving the quality of the data, and consequently the efficiency of the KDD process. Methods for data preparation involve operations of different types: data integration, data cleaning, data transformation and data reduction [16]. These operations are not exclusive since they may be combined. For example, data transformation can have an impact on data cleaning during normalisation of data. Data integration can have an impact on data cleaning as well, when inconsistencies are detected and corrected, or when missing values are filled. Still regarding data integration, the use of ontologies has been theoretically and practically studied in life sciences [17,18]. In this way, we have defined and used an ontology for integrating data on genetic variants [19]. Perez-Rey *et al*. have developed OntoDataClean, an ontology-based tool aimed at solving inconsistencies, missing and wrong values in datasets [20]. Data transformation operation produce formatted data, i.e. normalised and smoothed data, ready for being processed by data mining algorithms. Euler and Sholz propose a special ontology related to the transformation process [21]. This ontology provides facilities to manipulate data by using conceptualization of the transformation process.

The role of data reduction process is to reduce the description of data, e.g. lowering the number of dimensions within the data, without altering the integrity of the initial data set. Strategies for data reduction include the followings.

– **Data cube aggregation** produces data cubes for storing multidimensional aggregated data (e.g. extracted from a data warehouse) for OLAP analysis [22]. For example, data on daily sales hold on millions of items and can be aggregated into monthly sales of some selected categories of items.

– **Dimension reduction** leads to the encoding of data in a reduced format, with or without loss with respect to the initial data set. For example, principal component analysis can be used for dimensionality reduction that applies projections of initial data onto a space of a smaller dimension.

– **Data discretization** techniques are used to reduce the number of values of an attribute and consequently facilitate interpretation of mining results. Automatic discretization methods exist for continuous numerical attributes that recursively partition the attribute values according to a given scale. For example, the range of an attribute *price* can be divided by the means of histogram analysis into several intervals, which can in turn be iteratively aggregated into larger intervals. However, these methods do not apply for discrete or nominal attributes, when the attribute values of which are not ordered. The scale for an attribute has then to be manually defined by domain experts and possibly refined with the help of heuristic methods [23].

– **Data selection** aims at identifying appropriate subsets among the initial set of attributes. This operation can be performed with the help of heuristic methods based on tests of significance or entropy-based attribute evaluation measures such as the information gain [24,25]. Data selection is one of the data reduction methods that is studied in this paper.

The use of domain knowledge in KDD process can be considered from two points of view. The first one uses knowledge about the KDD process itself, i.e. domain represented within ontologies are data transformation, data cleaning, or the whole KDD domain [26]. The second one uses knowledge related to the dataset domain [18], e.g. pharmacogenomics. The work presented in this article follows the second view, and focuses on data preparation, and more precisely, on data selection. In addition it is made precise how available domain knowledge –contained in a knowledge base (KB)– can assist the domain expert in selecting relevant attributes or object subsets.

Our case-study deals with genotype-phenotype relationships. Finding relationships between genotype and phenotype is of primary interest in biological research. Large scale clinical studies provide large mass of genomic and post-genomic data produced by high-throughput biotechnology devices (e.g. microarray, mass spectrometry). Recent studies [27-29] have shown that data mining methods can be used for extracting unexpected and hidden correlations between genotype and phenotype. However, these studies also illustrate the difficulty of achieving these analyses, mainly because of domain complexity and large volume of data to be analysed. Keeping this in mind, we will illustrate here the benefits of using ontology for data selection within a KDD process, whose objective is to extract relationships between genomic variants and phenotype traits. The data sources explored in the experience described in this paper have two origins: (i) there are private datasets resulting from clinical investigations relative to Familial Hypercholesterolemia (FH), (ii) there are public databases (i.e. dbSNP, HapMap, OMIM, and Locus Specific Databases) partially integrated within SNP-KB, a knowledge-base developed in our laboratory. An example of expected relationships that can be of interest, is in concern with modulator variants, i.e. any genomic variant (or group of variants) related to disease or disease symptom modulation. Various levels of severity are for example observed in FH depending on allele versions of two genomic variants in the *APOE* gene (rs7412 and rs429358) [30]. Modulator variants are of particular interest in pharmacogenomics since they are known to modulate the metabolism and effect of drugs [31].

The next section on results presents an overview of the ontology-guided data selection method. Three scenarios of data selection are described and illustrate the proposition and its advantages.

## Results

### Overview

**Figure 1:**
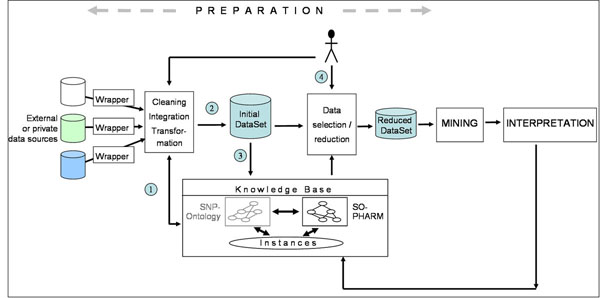
**Overview of the proposed method** The KDD process is divided into three main steps: data preparation, data mining, and data interpretation. The figure details data preparation within the KDD process and illustrates our method of data selection guided with domain knowledge. Data relevant to the study are collected from various resources such as genomic variation databases, published pharmacogenomic studies and private datasets. Various operations are applied to these data: cleaning, integration and transformation. Theses operations implies first an instantiation of a knowledge base (1), and second the design of the “initial dataset”(2). In this study, a dataset is defined as a relation between set of objects (rows) and set of attributes (columns). A mapping is then built between objects and attributes of this dataset and the instances from the KB (3). Data selection results from the definition of a subset of instances in the KB (4), allowing the selection of corresponding objects and attributes, with respect to the mapping. This process takes as inputs the initial dataset and the KB is controlled by the domain expert, and yields the “reduced dataset”. Characteristics of the ontology such as subsumption relationships, properties and class descriptions, are used to guide the definition of meaningful instance subsets. These subsets are in turn used for data selection. Data mining algorithms are then applied to the reduced dataset. The results of the mining operation are interpreted in terms of knowledge units that can be eventually integrated into the knowledge base.

An overview of the method is given in Figure 1. Data relevant to the study are collected from various resources such as genomic variation databases, published pharmacogenomic studies and private datasets. Various operations are applied to these data: cleaning, integration and transformation. These operations aimed first at participating in the instantiation of an existing KB, and second at producing the “initial dataset”. In this study, a dataset is defined as a relation between set of objects (rows) and set of attributes (columns). A mapping is then built between objects and attributes of this dataset, and instances of the KB. Data selection results from the definition of a subset of instances in the KB, allowing the selection of corresponding objects and attributes with respect to the mapping. This process that takes as inputs the initial dataset and the KB, is controlled by the domain expert, and yields the “reduced dataset”. Characteristics of the ontology such as subsumption relationships, properties and class descriptions, are used to guide the choice of meaningful instance subsets. These subsets are in turn used for data selection. Data mining algorithms are then applied to the reduced dataset. In the three examples presented hereafter, two mining algorithms are used. The first algorithm is Zart that extracts Frequent Itemsets (FI) and Frequent Closed Itemsets (FCI). The latter are special itemsets that cannot be extended in the dataset (see the Methods section). The ratio FI / FCI increases with the redundancy level of the itemsets. The second algorithm is COBWEB, which carries on a clustering of data in an unsupervised way. Actually, the results of the clustering are simply characterized by the number of obtained clusters.

### Articulation between data and knowledge

**Figure 2:**
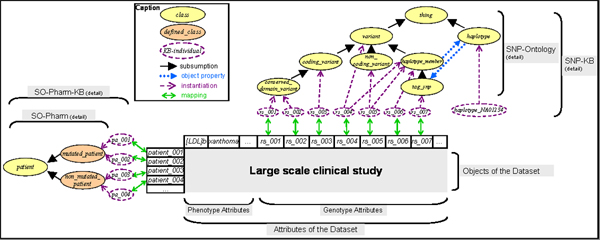
**Articulation between data and knowledge** Some classes of SNP- and SO-Pharm ontologies are shown as well as their assigned instances. The mapping between objects and attributes of the FH dataset, and instances of the KB is schematized.

Our method is based on a mapping between objects and attributes of the dataset, and instances of the KB. Thus, formalized knowledge within the KB can be used for guiding data selection. Figure 2 illustrates this mapping in the case of genomic variants assigned to concepts of the SNP-KB such as *conserved domain*_*variant*, *coding*_*variant*, *non*_*coding*_*variant*, *haplotype*_*member* or *tag*_*snp*.

The efficiency of the interaction between data and knowledge is mainly based on the instantiation process in the KB with collected data. This process is dependent on data integration issues and has to be controlled by the domain expert, who has to choose the most accurate class corresponding to the considered data. In this way, the domain expert is in charge of instantiating the right classes in the knowledge base. In practice, information about the mapping is stored in the KB during the instantiation process by adding a property to the created instance. It can be noticed that depending on modelling choices, one object or one attribute can be mapped to more than one instance. Three concrete scenarios for data selection are now described.

### Progressive selection of specific variants – guided by subsumption

The first scenario assumes that significant relationships between genotypes and phenotypes can be easily extracted from a reduced dataset, in which only coding variants or variants of conserved protein domains are considered. In our method, this kind of reduction results from the selection in the SNP-KB of a subset of instances corresponding to most specific and adequate classes in the ontology, with respect to subsumption relationships. As illustrated in Table 1, a progressive selection of the most specific variant instances, successively belonging to *variant* class and *coding*_*variant* and *conserved*_*domain*_*variant* subclasses, leads to a decreasing number of attributes related to variants in the dataset: progressively 289, 231, and 126 attributes. In practice, the guiding of instance selection is managed through a plug-in of Protégé 4 adapted for this purpose (see the Methods section).

**Table 1:** Quantitative characterization of data mining results depending on attribute selection. Table 1 gives quantitative information about output (number of itemsets and number of clusters) for two data mining methods involved in this experiment. A column corresponds to a various selection of attribute in the FH dataset.

	*variant*	*coding*_*variant*	*conserved*_*domain*_*variant*	*tag*_*snp*
Number of Variants	289	231	126	198

FI (FCI) {ratio FI/FCI}	6928 (255) {27. 17}	314 (24) {13.08}	304 (12) {25.33}	300(28){10.71}
Clusters	194	186	56	40

Table 1 shows in addition the amount of data mining results obtained when most specific classes of variants are selected. When all variants are considered (*variant* column), the total number of FI computed by Zart is 6928. With COBWEB, the total number of clusters is 194. At present, these results are complex to interpret due to the large volume of involved variants and the lack of contextual data. For example, coding and non coding variants cannot be distinguished.

The volume of data mining results progressively decreases as more reduced sets of variants are selected (*coding*_*variant* and *conserved*_*domain*_*variant* columns). This reduction can be read on the number of FI –from 6928 to 304– and of clusters –from 194 to 56– making results easier to interpret.

Being able to use subsumption relationships between ontology classes for guiding data selection is one main advantage resulting from the knowledge formalization effort, data integration and data cleaning preceding the SNP-KB instantiation.

### Tag-SNP based variant unification – guided by object properties

The examination of the data mining results obtained with the complete variant dataset reveals a high proportion of trivial and redundant association rules. This reflects the existence of variants belonging to the same haplotype. In simple words, ahaplotype designates a group of variants that segregate uniformly and can be replaced by a smaller group of variant, called “tag-SNPs”. Replacing all members of a haplotype by corresponding tag-SNP(s) may lower the number of extracted redundant association rules.

**Figure 3:**
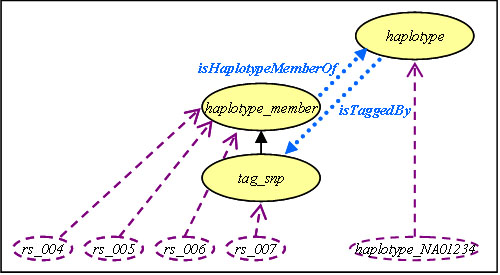
**Tag-SNP variant unification**. This figure focuses on some classes and instances from Figure 2. It develops the description of *Haplotype* and the *isHaplotypeMemberOf* and *isTaggedBy* object properties used for illustrating functional dependencies between instances of *variants* and *tag*_*snp*.

Figure 3 shows a haplotype composed of variants *rs*_*004*, *rs*_*005*, *rs*_*006* and *rs*_*007*, that can be replaced by the unique *rs*_*007* tag-SNP. This information, which actually depends on the description of a given haplotype (NA01234), enlightens a functional dependency between variant *rs*_*004* (or *rs*_*005* or *rs*_*006*) and *rs*_*007*. Such a functional dependency can be expressed in the SNP-knowledge base as follows.

*rs*_*004* := ***isHaplotypeMemberOf****(haplotype*_*NA01234)*

*rs*_*004* := ***isHaplotypeMemberOf****(**isTaggedBy** (rs*_*007))*

A knowledge base may include information about functional dependencies taking the form of object properties (or sequences of object properties). Since the SNP-KB includes haplotype descriptions issued from the HapMap project [32] and Haploview software [33], and includes *isHaplotypeMemberOf* and *isTaggedBy* properties, then it is possible to distinguish between tag-SNPs and other haplotype members in the SNP-KB. According to our method, reducing the dataset to tag-SNPs is based on the selection of a subset of variant instances of the *tag_snp* class. In the situation depicted in Figure 3, this implies in turn the removal of columns *rs*_*004*, *rs*_*005*, and *rs*_*006* in the dataset.

Applied to the FH initial dataset, this strategy considerably reduces the number of attributes (see Table 1, compare the *variant* and *tag*_*snp* columns). The volume of extracted units to be interpreted is thus also considerably reduced, not only because of the lower number of attributes but also because of the reduced number of dependencies between selected attributes (see the percentage of non redundant rules). One main advantage of guiding this selection process with domain ontology is to dynamically use the representation of functional dependencies between simple haplotype members and representative tag-SNPs in the SNP-KB. The representation is dependent on the precision of haplotype construction and may evolve. Automated updating of haplotype representation and instantiation in the SNP-KB is under study.

### Patient selection – guided by class definition and classification

In contrast with the two previous scenarios dedicated to attribute selection, e.g. *variant*, this paragraph illustrates object selection, e.g. *patient* selection, leading to a reduction of the dataset as well. This third scenario illustrates the selection of instances based on the description of classes within SO-Pharm ontology. SO-Pharm encompasses and extends SNP-Ontology (see Methods section).

In the FH case study, groups of patients suspected to present specific genotype-phenotype profiles are defined. Classes and properties of SO-Pharm allow to define four classes of patients: one already existing in SO-Pharm, and three others that are defined for the data selection.

*patient* (defined in SO-Pharm)

*patient*_α ≡ *patient* ⊓ ∃ *presentsGenotypeItem (*∋ *(LDLR*_*mutation))*

*patient*_β ≡ *patient* ⊓ ∃ *presentsGenotypeItem (*∋ *(no*_*LDLR*_*mutation))*

⊓ ∃ *presentsPhenotypeItem (*∋ *(high*_*LDL*_*in*_*blood))*

*patient*_γ ≡ *patient* ⊓ ∃ *presentsGenotypeItem (*∋*(no*_*LDLR*_*mutation))*

⊓ ∃*presentsPhenotypeItem (*∋*(normal*_*LDL*_*in*_*blood))*

Reasoning mechanisms as applied to instances classify patients according to their individual properties. This allows to detect and to select a set of objects sharing the same attributes, as a set of instances belonging to the same class. This selection may reduce the volume of data input for subsequent mining tasks, and allows the characterization and comparison of selected subgroups.

## Discussion

Data selection is a crucial step in KDD process and any attention paid to selection makes more efficient the KDD process. Indeed, the computational cost in space and time of data mining algorithms is exponential (at worst), and any reduction of the initial dataset has effect on the whole data mining process. In addition, the practical use of data mining algorithms is also often limited by size of datasets or machine capabilities. For example, the extraction of frequent itemsets from the FH dataset on a standard workstation with a Pentium 1.8Ghz and 2Mb of RAM has to be limited to the calculation of the “most frequent” itemsets since the minimum support has to be set very high (i.e. 96%). Data selection is an important operation participating to the preparation step of the KDD, allowing the data mining algorithm to handle large dataset. Comparative tests show that data selection reduces quite always the volume of results and, in some cases, the redundancy within the extracted units. The efficiency of data selection is not so surprising and demonstrates, to a certain extent, some advantages of using ontology. More importantly, an actual positive feedback from the domain expert has been observed, who has enthusiastically piloted the data selection, being assisted by an ontology. The smaller size of the results has been a second cause of satisfaction for the domain expert, since results of the data mining tests have revealed non-standard results that may be of interest with respect to the domain knowledge.

Ontology-guided data selection can be performed by taking advantage of subsumption relationships between ontology classes and by defining subsets of instances corresponding to the most specific classes. When association rules have been extracted from a reduced dataset, the subsumption relationships can be followed within the ontology, for generalizing the association rules. This bottom-up traversal of the ontology can be used, for example, to check whether an extracted association rule between a coding variant and a phenotypic trait can be extended to some non-coding variants. This kind of association may be observed when intron splice sites are affected as discussed in [34].

## Conclusions

This paper illustrates how domain knowledge captured in bio-ontologies facilitates the KDD process. An approach for data selection has been proposed that takes good advantage of time and effort spent for the KB construction.

Three proposed scenarios of data selection can be combined in order to define optimized KDD strategies fulfilling biomedical objectives. For that purpose, additional scenarios can be planned such as object unification, i.e. grouping together patients from the same family and retaining a unique representative for the family, thus reducing the number of objects to be manipulated. The selection process depends on instance properties (object and data properties), and accordingly on data and instantiation quality. When an instance is missing or presents a fault, the selection will be erroneous or impossible. In this way, the available knowledge on haplotypes could also be used for completing missing values about observed alleles of each member of a haplotype.

Challenging future work consists in automatically formalizing the results of the KDD process within a knowledge representation language, for enriching both the ontology and the KB. Such a capability allows to iteratively run the KDD process, using more complete domain knowledge after each KDD iteration.

## Methods

### The FH dataset

Objects in the FH datasets are patients of a clinical study related to Familial Hypercholesterolemia. Attributes are data relative to the phenotype or the genotype of the patients.

The dataset concerns:

(α) patients affected by the genetic hypercholesterolemia (FH),

(β) patients affected by a non-genetic hypercholesterolemia, and

(γ) patients without any hypercholesterolemia.

Majority of genotype attributes (289/293) describes observed alleles for genomic variants of the *LDLR* gene. An example of genotype attribute is the observed allele for the variant located at position Chr19:11085058 (e.g. AA). Phenotype attributes describe traits usually observed when studying the metabolism of lipids. Two examples of phenotype attributes are the LDL blood concentration (e.g. [LDL]_b_=3gl^−1^) and the presence/absence of xanthoma. Table 2 describes quantitatively the dataset.

**Table 2:** Characteristics of the FH dataset. The FH dataset results from a clinical study relative to Familial Hypercholesterolemia. Its size and composition are described in Table 2. Phenotype refers to phenotypic attributes including for instance LDL concentration in blood. Genotype attributes include 289 genomic variations of the *LDLR* gene and 3 attributes relative to the presence of mutations in 3 other genes.

Objects	*Patients*		125
Attributes	*Phenotype*	12	304

	*Genotype*	292	

### SNP-Ontology and SO-Pharm

The SNP-Ontology [35] includes a formal representation in OWL-DL (i.e. the Ontology Web Language) of genomic variations and their related concepts: sequence in which they are observed, haplotype they belong to, proteins they modify, database in which they are stored, etc. For this study, a SNP-Knowledge Base (SNP-KB) is populated according to the semantic structure of the SNP-Ontology and integrating knowledge about genomic variations of the *LDLR* gene (Figure 2). Partially integrated data sources are dbSNP, HapMap, OMIM and private or public Locus Specific Databases [36]. The method used to populate the SNP-KB is described precisely in [19].

SO-Pharm is an OWL-DL ontology embedding knowledge about clinical studies in pharmacogenomics [37,38]. SO-Pharm satisfies all quality principles defined by the OBO Foundry [39]. It is closely articulated with the SNP-Ontology as with other ontologies that include knowledge about other pharmacogenomics sub-domains, i.e. related to drug, genotype, and phenotype. SO-Pharm and articulated ontologies are used to guide the data selection process.

### Knowledge management and instance selection tools

Instantiation of classes in the ontologies is managed both with Protégé [40] and Jena API [41]. Consistency checking and classification are carried on with Pellet 1.4 [42]. Practically, the instance selection is performed through an adapted Protégé 4 plug-in [43]. This plug-in allows the selection of instances sharing characteristics, e.g. class membership, properties, relation with another specific instance, (a) by browsing and selecting items in hierarchies of classes, object properties and list of instances in a KB, (b) by answering DL queries with complex restrictions. This plug-in is currently under development and is planned to be released in a near future for the scientific community.

### Data mining methods

Data mining tests have been run on the FH dataset with two different unsupervised algorithms. The first one, named Zart, extracts association rules after searching for frequent itemsets [44,45]. Zart generates itemsets of the form “*ABC*” from which in turn is derived an association rules such as “*AB implies C*”. An itemset is characterized by its support, i.e. the frequency of its occurrence in the dataset. Frequent Itemsets (FI) are itemsets with a support greater to a minimum threshold or minimum support, which has to be fixed by the domain expert. Frequent Closed Itemsets (FCI) are FI having the characteristic of not being included in any superset, i.e. a larger itemset, with the same support. Zart has been parameterized with a minimum support of 96% for the experiment. The principal motivation for using Zart is that this algorithm generates FI, FCI, and in addition, the so-called minimal generators allowing to infer the set of minimal non-redundant association rules. COBWEB is a second algorithm designing a structural clustering [46]. COBWEB is parameterized with an acuity=1 and a cutoff=0.5 that affect the construction of clusters with constraints on their relation and their cardinality. COBWEB is an algorithm of interest in the present study, because it generates a cluster hierarchy that can be reused in parallel with FI and FCI (the use of these clusters is planned in a future work).

The implementations of Zart and COBWEB mentioned just before are available respectively in the Coron platform [47] and the Weka toolbox [48].

## List of abbreviations used

API – Application Programming Interface

dbSNP – Single Nucleotide Polymorphism database

DL – Description Logics

FCI – Frequent Closed Itemset

FH – Familial Hypercholesterolemia

FI – Frequent Itemset

KB – Knowledge Base

KDD – Knowledge Discovery in Database

LDL – Low-Density Lipoprotein

*LDLR* – Low-Density Lipoprotein Receptor

NCBO – National Center for Biomedical Ontology

OBO – Open Biomedical Ontologies

OLAP – Online Analytical Processing

OMIM – Online Mendelian Inheritance in Man

OWL – Web Ontology Language

RAM – Random Access Memory

SNP – Single Nucleotide Polymorphism

## Competing interests

The authors declare that they have no competing interests.

## Authors' contributions

AN carried the initial purpose of using domain knowledge in KDD process. AC, MS, MDD designed the method. AC implemented the framework and performed tests. PB carried out the FH clinical study and analyse data selection and data mining results. AC, MS, AN, MDD contributed to write the manuscript. All authors read and approved the final manuscript.
